# Supplementary material for: The MADS-Box Transcription Factor EjAGL18 Negatively Regulates Malic Acid Content in Loquat by Repressing EjtDT1
Source: Int J Mol Sci. 2025 Jan 10;26(2):530. doi: 10.3390/ijms26020530 (PMC11765138; doi:10.3390/ijms26020530)
Supplement: Supplementary file 1 [file ijms-26-00530-s001.zip › Figures.pdf]

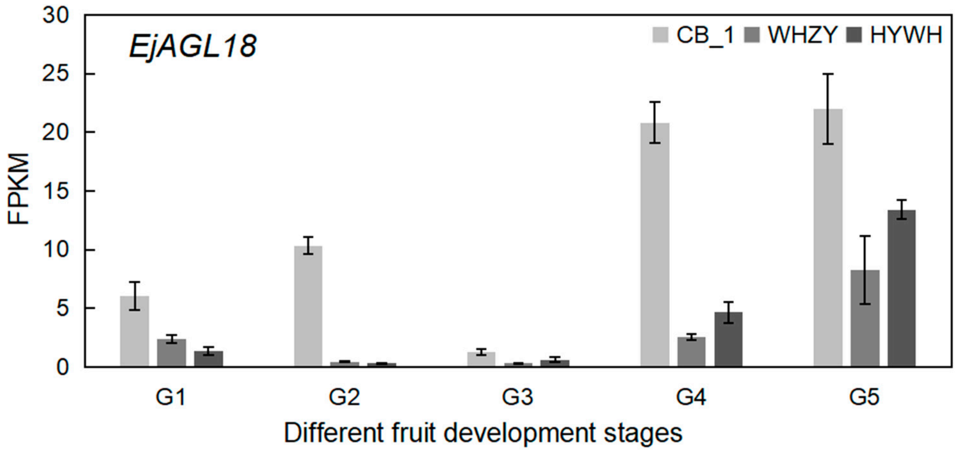

Figure S1. The transcriptional level of *EjAGL18* in loquat fruits of different development stages. 'CB_1', 'WHZY' and 'HYWH' represented 'Changbai No.1', 'Wuhe Zaoyu' and 'Huayu Wuhe No.1', respectively. The data of this bar chart originates from our previous transcriptome data (Chi et al., 2023). G1-G5 represented different development stages of loquat fruits. The values are shown as the mean ± SE.

Chi, Z.H.; Liu, X.Y.; Wen, S.Q.; Wang, Y.; Lv, W.J.; Guo, Q.G.; Xia, Y.; Jing, D.L.; Liang, G.L. Integrated metabolomic profiling and transcriptome analysis of fruit quality and ripeness in early-maturing seedless triploid loquat. *Sci. Hortic.* **2023**, *316*. https://doi.org/10.1016/j.scienta.2023.112012.

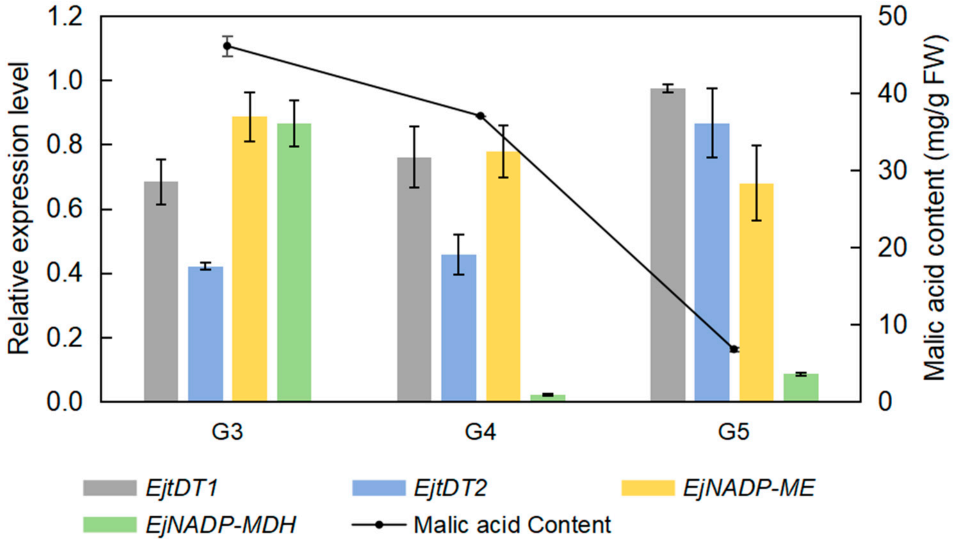

Figure S2. The expression patterns of *EjtDT1/2* and *EjNADP-ME/MDH* and the accumulation pattern of malic acid in loquat fruits at different development stages. The genes expressions were shown as bar charts and the malic acid content was shown as line graph. G3-G5 represented three development stages of loquat fruits. The values are shown as the mean ± SE.

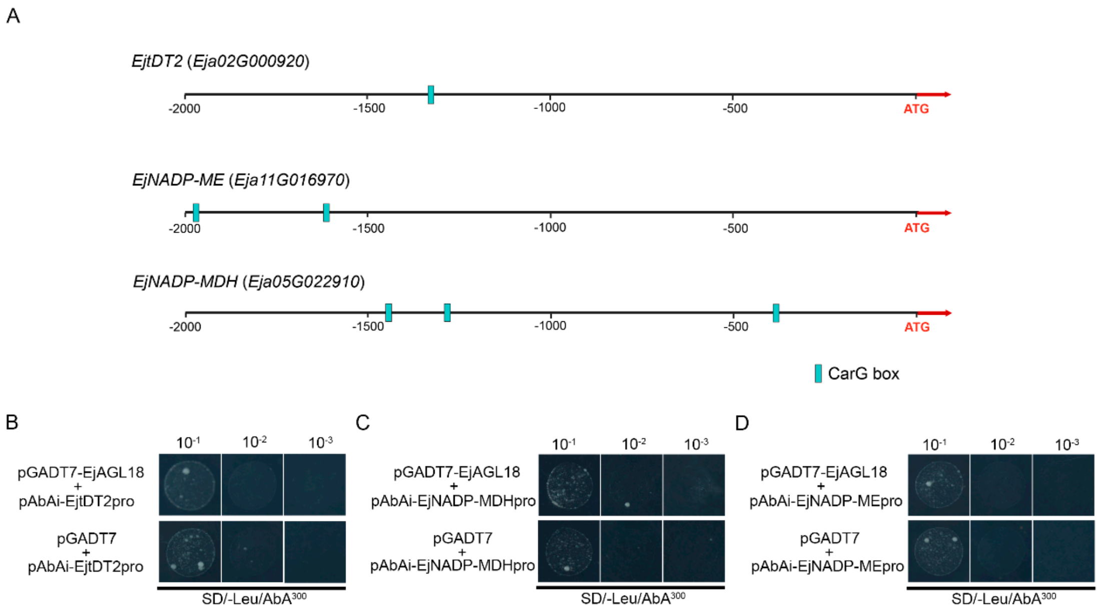

Figure S3. EjAGL18 did not bind to the promoters of *EjtDT2*, *EjNADP-ME* and *EjNADP-MDH*. (**A**) The CarG box sites in the promoters of *EjtDT2*, *EjNADP-ME* and *EjNADP-MDH*. (**B**) Y1H assay of EjAGL18 and *EjtDT2* promoter. Y1H assay. The co-transformation yeast cells of empty vector pGADT7 along with *EjtDT2* promoter (pGADT7 + pAbAi-EjtDT2pro) were used as controls. (**C**) Y1H assay of EjAGL18 and *EjNADP-MDH* promoter. The co-transformation yeast cells of empty vector pGADT7 along with *EjNADP-MDH* promoter (pGADT7 + pAbAi-EjNADP-MDHpro) were used as controls. (**D**) Y1H assay of EjAGL18 and *EjNADP-ME* promoter. Y1H assay. The co-transformation yeast cells of empty vector pGADT7 along with *EjNADP-ME* promoter (pGADT7 + pAbAi-EjNADP-MEpro) were used as controls. The screened concentration of ABA was 300 ng/mL.
